# Exploring new frontiers in oncofertility preservation: a case of ovarian stimulation during pregnancy

**DOI:** 10.1186/s13048-025-01615-4

**Published:** 2025-02-26

**Authors:** Parisa Pirooznia, Esmat Mashhadi Meighani, Firouzeh Ghaffari

**Affiliations:** Department of Endocrinology and Female Infertility, Reproductive Biomedicine Research Center, Royan Institute for Reproductive Biomedicine, ACECR, Tehran, Iran

**Keywords:** Pregnancy-associated breast cancer, PABC, Fertility preservation, Random start ovarian stimulation

## Abstract

**Background:**

The standard treatment for Pregnancy-Associated Breast Cancer (PABC) includes surgery and neoadjuvant chemotherapy, which can impair fertility, emphasizing the critical need for fertility preservation in these patients. This case report discusses a breast cancer patient who was found to be pregnant shortly after starting treatment. Despite the pregnancy and increased levels of βHCG and progesterone, the ovarian stimulation cycle yielded a satisfactory number of mature oocytes and high-quality embryos.

**Case presentation:**

A 40-year-old woman, G1Ab1 (Gravida1Abortion1), who was diagnosed with Invasive Ductal Carcinoma with negative receptors (Estrogen Receptor, Progesterone Receptor, and Human Epidermal Growth Factor Receptor 2) was referred to the oncofertility unit of the Royan Infertility Center for fertility preservation prior to the commencement of chemotherapy. Following necessary consultations and procedures, and confirming a negative pregnancy test, a random start letrozole-based protocol was initiated for ovarian stimulation. During the cycle, a positive pregnancy test was encountered. Despite the positive test, the cycle continued, and on day 13 of the cycle, triggering was performed with a GnRH agonist. A puncture was performed 36 h later, yielding 12 oocytes and 8 embryos.

**Conclusion:**

This case highlights the feasibility of adapting random-start ovarian stimulation protocols during pregnancy, warranting further investigation in similar clinical scenarios.

## Background

Emerging research delineates that approximately 20% of breast cancer diagnoses in individuals under the age of 30 are concomitant with pregnancy. The malignancies identified during gestation frequently exhibit advanced stages or poor differentiation [1–3]. Consequently, the initial therapeutic interventions for Pregnancy-Associated Breast Cancer (PABC) include surgical intervention, with chemotherapy administered either preoperatively or postoperatively. These intensive and gonadotoxic treatments may precipitate fertility impairment, underscoring the imperative need for fertility preservation strategies in PABC patients [1–3].

Contemporary approaches to fertility preservation in female oncology patients incorporate oocyte, embryo, and ovarian tissue cryopreservation. However, in scenarios necessitating immediate commencement of cytotoxic drug therapy, sufficient time to complete the ovarian stimulation cycle may be lacking. This presents a significant challenge in preserving fertility in female oncology patients, particularly concerning the timing of initiating the controlled ovarian hyperstimulation (COH) cycle [1–3].

In light of these challenges, random start COH has been proposed in recent years. Given that this protocol can be initiated at any point during the menstrual cycle, potential pregnancy must be taken into account. As chemotherapy is contraindicated during the first trimester of gestation, if pregnancy coincides with breast cancer and necessitates chemotherapy, pregnancy termination may be required [1].

In this context, we report a case of a breast cancer patient who underwent an ovarian stimulation cycle as a fertility preservation option prior to the commencement of chemotherapy. Subsequent to the initiation of the ovarian stimulation protocol, the patient was diagnosed with pregnancy. Notwithstanding the concurrent pregnancy and elevated levels of βHCG and progesterone, a satisfactory quantity of M2 and high-quality embryos were produced.

## Case presentation

A 40-year-old female patient who was referred to the oncofertility preservation unit of the Royan Institute on December 19, 2022, due to a breast cancer diagnosis and the subsequent need for fertility preservation. The patient, who had been married for two years and had a regular menstrual cycle (with a periodicity of 30 days), had previously experienced a miscarriage (blighted ovum) in the preceding year.

After detecting a lump in her left breast, sonography revealed a heterogeneous 27 × 16 mm lesion at the 1–2 o’clock position (B5) and two suspicious left axillary lymph nodes (9.5 mm, B4) [4]. A core needle biopsy from the left breast revealed Invasive Ductal Carcinoma (stage CT2M1) [4]. The Estrogen Receptor (ER), Progesterone Receptor (PR), HER2, and P63 were all negative, while Ki67 was reported at 40–45%.

Based on the oncologist’s evaluation, the patient’s diagnosis of breast cancer necessitated the administration of chemotherapy. Given the known risk of chemotherapy-induced ovarian failure, the patient was identified as a candidate for fertility preservation strategies. Preceding the commencement of chemotherapy, she was referred to a specialized fertility center for the implementation of fertility preservation techniques. A comprehensive consultation session was conducted, during which both the patient and her spouse engaged in detailed counseling with an infertility specialist regarding the implications and options for fertility preservation. Following this discussion, and with the informed consent of the couple, the technique of embryo cryopreservation was elected as the fertility preservation method.

The following table (Table 1) chronologically outlines the case management, integrating cycle days, sonographic and laboratory data, clinical decisions, and outcomes, highlighting the coordination between fertility preservation team and oncologic care as a multidisciplinary team.

**Table 1:** Chronological timeline of clinical management and outcomes in fertility preservation via ovarian stimulation during pregnancy

Cycle Day	Key Actions/Decisions	Sonographic Findings	Lab Results	Rationale/Outcome
(Menstrual day) Day 28	Referral to the infertility center; initial tests were ordered.	-	βhCG: 4 mIU/mL Estradiol: 2.6 pg/mL Progesterone: 15.3 ng/mL FSH: 2.4 mIU/mL LH: 4.94 mIU/mL AMH: 5.32 ng/mL	Pregnancy was ruled out.
Day 29 (Stimulation Day 1)	Transvaginal sonography was performed post-negative βhCG. Ovarian stimulation was initiated: Letrozole (5 mg) + Follitropin alfa (150 IU).	Right ovary AFC: 10 Left ovary AFC: 20 Endometrial thickness: 7 mm	-	Ovarian reserve was confirmed (AFC 30 total); ovarian stimulation was planned. Given the concern regarding estrogen’s potential deleterious impact on oncological progression, a regimen combining letrozole with gonadotropins was prescribed for the ovulation cycle.
Stimulation Day 5	Poor follicular response led to dose escalation.	No follicular response was observed in either ovary.	Estradiol: 55 pg/mL	Follitropin alfa was increased to 225 IU and HMG 75 IU were added alongside with letrozole 5 mg.
Stimulation Day 7	Stimulation was continued.	Follicles: 8–10 mm were observed in both ovaries.	Estradiol: 80 pg/mL	Doses were adjusted: Follitropin alfa 225 IU + HMG 150 IU + letrozole 5 mg
Stimulation Day 9	Follicular growth was monitored.	Right ovary: 1 follicle (12 mm) Both ovaries: 3 follicles (11 mm), 5 follicles (10 mm), 3 follicles < 7 mm	Estradiol: 140 pg/mL	Stimulation was continued.
Stimulation Day 10	GnRH antagonist (Cetronax 0.2 mg) was started; βhCG was detected.	-	βhCG: Positive The βhCG (beta-human chorionic gonadotropin) test was conducted as a routine preliminary assessment prior to the PET scan.	Pregnancy was confirmed.
Stimulation Day 12	Follicles were monitored.	Largest follicles: 14–16 mm Endometrial thickness: 12 mm	Estradiol: 802 pg/mL Progesterone: 15.3 ng/mL LH: 0.4 mIU/mL βhCG: 230 mIU/mL	-
Stimulation Day 13	GnRH agonist trigger (Superfact 1 cc) was administered.	Follicles: 7 follicles > 18 mm, 4 follicles > 14 mm	βhCG: 372 mIU/mL Estradiol: 848 pg/mL	Oocyte maturation was triggered for retrieval. multidisciplinary decision was made to terminate.
Stimulation Day 15	Oocyte retrieval + suction curettage were performed.	Endometrial thickness: 15 mm Pregnancy sac: 4 mm (intrauterine)	βhCG: 987 mIU/mL	12 oocytes were retrieved (5 mature). 8 embryos were cryopreserved. Pregnancy was terminated. Chemotherapy was commenced the following day.
Post-Day 15	Patient was discharged; follow-up was conducted.	-	βhCG: Negative (8 days post-curettage)	Safe discharge was ensured. To mitigate the risk of developing Ovarian Hyperstimulation Syndrome (OHSS), GnRH antagonists were prescribed for a duration of 8 days following the patient’s discharge

Table Captions: AFC: Antral Follicle Count; AMH: Anti-Müllerian Hormone; βhCG: Beta Human Chorionic Gonadotropin; COH: Controlled Ovarian Hyperstimulation; ER: Estrogen Receptor; FSH: Follicle-Stimulating Hormone; GnRH: Gonadotropin-Releasing Hormone; GV: Germinal Vesicle; HER2: Human Epidermal Growth Factor Receptor 2; HMG: Human Menopausal Gonadotropin; IVM: In Vitro Maturation; LH: Luteinizing Hormone; M2: Metaphase II; OHSS: Ovarian Hyperstimulation Syndrome; PET Scan: Positron Emission Tomography Scan; PR: Progesterone Receptor

On the day of oocyte retrieval (day 15), a transvaginal sonography was performed. The endometrium was thick and measured at 15 mm, and a pregnancy sac with a diameter of 4 mm was observed in the uterus. Thirty-six hours after the trigger, oocyte retrieval was performed with 22 follicles punctured; meanwhile, βHCG was reported as 987 mIU/ml. A total of twelve oocytes were obtained. Of these, five were mature M2 oocytes. Post-intracytoplasmic sperm injection (ICSI), these five oocytes were fertilized, resulting in five zygotes denoted as 5(2PN). Subsequently, these zygotes developed into five cleavage-stage embryos with the following grades: 4AB, 4B, 2AB, 2B, and 3B [5]. In addition, six oocytes were identified at the germinal vesicle (GV) stage and subjected to an in vitro maturation (IVM) culture protocol. This IVM process culminated in the maturation of three additional M2 oocytes. Following ICSI, these oocytes were fertilized, yielding three zygotes (3(2PN)) and subsequently three embryos with the grades 8AB, 8AB, and 8BC. However, three oocytes within the GV-stage group failed to reach maturation, and one oocyte was non-viable post-retrieval. Ultimately, eight cleavage stage embryos were cryopreserved. On the day of retrieval, suction curettage was performed to terminate the pregnancy due to the necessity for chemotherapy and the prohibition of continuing the pregnancy. Three hours post-procedure, the patient was discharged from the institute without any complications. And chemotherapy was started. Eight days post-curettage, βHCG levels returned to negative.

## Discussion

The random start ovarian stimulation protocol is employed for oocyte and embryo cryopreservation for fertility preservation in cancer patients who lack sufficient time to perform routine ovarian stimulation at the onset of the menstrual cycle. This protocol can be executed at any point during the cycle. Evidence suggests that the random start protocol offered to cancer patients shares a similar outlook as conventional IVF methods. According to a study conducted by Koang et al., the pregnancy rate following stimulation initiation in the luteal phase is akin to stimulation initiation in the early follicular phase [6]. A systematic review and meta-analysis found that the clinical pregnancy rate (CPR) and live birth rate (LBR) were identical following follicular and luteal phase stimulation [7].

The required drug dose for stimulation in the luteal phase is slightly higher than when starting at the beginning of the follicular phase. To mitigate increased estrogen concentration during the ovulation stimulation cycle, it is recommended to add aromatase inhibitors like letrozole at the beginning of the stimulation cycle in estrogen sensitive cancers such as breast cancer and endometrial adenocarcinoma [2, 3, 8].

One of the recommended regimens in random start ovarian stimulation is the use of progestins, a protocol known as Progesterone Primed Ovarian Stimulation (PPOS). Progesterone, a crucial regulator during ovulation, can be utilized in lieu of GnRH antagonist to prevent premature luteinization. When progestins are prescribed in the early stages of the cycle, prior to an increase in estrogen, they suppress GnRH secretion in the hypothalamus. It has been demonstrated that LH levels exhibit greater stability with PPOS. However, due to premature exposure to progesterone and the resultant asynchrony between embryo growth and endometrial receptivity, embryos obtained in that cycle are not transferred [6, 9]. This protocol is deemed appropriate in scenarios where embryo transfer is not necessary, including ovarian stimulation in egg donors, fertility preservation, and cycles that necessitate preimplantation genetic testing (PGT) [10]. The majority of studies indicate that outcomes related to ovulation response, such as the gonadotropin dose, the number of retrieved oocytes, and the mature (M2) oocytes count, are comparable between the PPOS protocol and GnRH agonist cycles [11]. In a meta-analysis conducted by Guan et al., it was shown that the CPR, LBR, and ongoing pregnancy rate were comparable in the PPOS protocol relative to other ovulation cycles. The PPOS protocol also reduces the likelihood of Ovarian Hyperstimulation Syndrome (OHSS) occurrence in patients with Polycystic Ovary Syndrome (PCOS), both in the follicular and luteal phases [12]. Due to better control of LH concentration, lower drug costs, easier administration (oral), and comparable pregnancy and neonatal outcomes with regular cycles, PPOS has been considered as a flexible protocol [6, 9, 12]. In a randomized clinical trial, the PPOS protocol was unexpectedly associated with a reduced LBR in comparison to cycles using a GnRH antagonist protocol [13]. In this case, given the temporal limitations, a random start protocol was employed to expedite ovulation induction. Notably, the inadvertent conception and the resultant elevation in progesterone levels paralleled the physiological responses observed in PPOS cycles, thereby fulfilling the intended benefits of the protocol.

We postulate that in the aforementioned patient, the elevated level of endogenous progesterone following pregnancy led to the suppression of FSH and LH. Consequently, the dose of gonadotropin used in the ovulation stimulation cycle was increased. In PPOS cycles, high progesterone levels contribute to better LH stability, and it appears that the increase in endogenous progesterone has played a role in enhancing the quality of oocytes and embryos through this mechanism. Additionally, the risk of OHSS in PPOS cycles is lower, and our patient, despite having a high AFC and AMH equal to 5.3 ng/ml, did not develop OHSS due to receiving a high dose of gonadotropin through this mechanism. Moreover, elevated HCG levels can increase the risk of OHSS. A case of OHSS following a PPOS cycle due to an ectopic pregnancy during ovarian stimulation has been reported [14]. In our patient, HCG levels were found to be low. We undertook the actions of simultaneous pregnancy termination and oocyte retrieval in the presence of elevated progesterone levels, which successfully prevented the development of OHSS. One of the risk factors for OHSS in oncology patients is the use of long-acting GnRH agonists recommended prior to initiating chemotherapy. The administration of these agonists with a short interval following oocyte retrieval is intended to suppress the menstrual cycle and offer ovarian protection. However, this can lead to an increase in FSH, LH, and estradiol levels, which can mimic the physiological effects of HCG, thereby potentially precipitating OHSS [15]. It seems plausible that if future studies can independently confirm the sufficiency of endogenous progesterone in controlling LH surge under these conditions, eliminating antagonist in ovulation stimulation cycle will be both patient-friendly and cost-effective.

The presence of a measurable level of HCG at the beginning of the ovulation stimulation cycle presents a challenging issue. The increasing interest in the role of HCG on oocyte quality has stemmed from case reports on unintentional stimulation in previous pregnancies. The impact of HCG on oocyte quality remains a contentious issue. Some studies have suggested that HCG likely reduces oocyte quality by causing early luteinization. Conversely, other studies have proposed that the presence of low-level HCG during the ovulation stimulation cycle enhances granulosa cell sensitivity to gonadotropins, ultimately increasing mature dominant oocytes and quality embryos. A possible explanation for these divergent findings could be attributed to different HCG levels in these studies [1, 16].

In our patient, the βHCG level was reported to be as high as 372 on the trigger day and 987 mIU/ml on the puncture day. It appears that due to the low level of βHCG, it did not negatively impact oocyte quality. On the other hand, the increase in HCG level from 372 to 987 mIU/ml on the puncture day may have acted as a trigger. Along with GnRH agonist administration, this could have had dual trigger effects on oocyte maturation and aided in better maturation of oocytes [17]. Unlike previous studies, which typically initiated fertility preservation after pregnancy termination or delivery, this case proceeded with ovarian stimulation concurrently with pregnancy. A 2021 case report described ovarian tissue cryopreservation in a pregnant breast cancer patient, but tissue collection occurred after cesarean section at 32 weeks, necessitating pregnancy termination before ovarian tissue cryopreservation [18].

Despite the formation of eight embryos of acceptable quality, due to the incomplete treatment cycle of the patient and inability to transfer embryos, her pregnancy outcome remains unknown. Further research and reports in this area would be beneficial for better selection of ovulation stimulation cycle type in similar patients.

### Limitations and future directions

Small Sample Size: As a single-case report, broader validation is needed by further studies.

Long-Term Outcomes: The patient’s future pregnancy potential using cryopreserved embryos remains unknown.

## Conclusion

This case demonstrates the technical feasibility of ovarian stimulation during pregnancy for oocyte retrieval, though efficacy and safety require validation in larger cohorts. This approach may be particularly advantageous for cancer patients who are unable to delay fertility treatments due to their condition and are candidates for gonadotoxic treatment, where the opportunity for preserving fertility does not exist. It suggests that ovarian stimulation can be integrated into urgent cancer care timelines, even with concurrent gestation. While the successful retrieval of oocytes during pregnancy in this instance is noteworthy, it does not constitute an endorsement of this approach as a conventional protocol for pregnant individuals. Prior to any global recommendation, extensive research and a thorough examination of ethical standards are imperative.

## Data Availability

No datasets were generated or analysed during the current study.

## Abbreviations

2PN: Two pronuclei
AFC: Antral follicle count
AMH: Anti-mullerian hormone
βHCG: Beta-human chorionic gonadotropin hormone
COH: Controlled ovarian hyperstimulation
CPR: Clinical pregnancy rate
ER: Estrogen receptor
FSH: Follicle-stimulating hormone
G1Ab1: Gravida1Abortion1
GnRH: Gonadotropin-releasing hormone
GV: Germinal vesicle
HER2: Human epidermal growth factor receptor 2
ICSI: Intracytoplasmic sperm injection
IVF: In vitro fertilization
IVM: In vitro maturation
LBR: Live birth rate
LH: Luteinizing hormone
M2: Metaphase II
OHSS: Ovarian hyperstimulation syndrome
PABC: Pregnancy-associated breast cancer
PCOS: Polycystic ovary syndrome
PGT: Preimplantation genetic testing
PET scan: positron emission tomography scan
PPOS: Progesterone primed ovarian stimulation
PR: Progesterone receptor
